# Percutaneous MR-guided focal cryoablation for recurrent prostate cancer following radiation therapy: retrospective analysis of iceball margins and outcomes

**DOI:** 10.1007/s00330-017-4833-9

**Published:** 2017-04-13

**Authors:** Christiaan G. Overduin, Sjoerd F. M. Jenniskens, J. P. Michiel Sedelaar, Joyce G. R. Bomers, Jurgen J. Fütterer

**Affiliations:** 1 Department of Radiology and Nuclear Medicine, Radboud University Medical Center, P.O. Box 9101 (767), 6500 HB Nijmegen, The Netherlands; 2 Department of Urology, Radboud University Medical Center, Nijmegen, The Netherlands; 3 MIRA Institute for Biomedical Engineering and Technical Medicine, University of Twente, Enschede, The Netherlands

**Keywords:** Ablation margin, Cryoablation, MRI, Prostate cancer, Local recurrence

## Abstract

**Objectives:**

To evaluate iceball margins after magnetic resonance (MR)-guided focal salvage prostate cryoablation and determine the correlation with local outcome.

**Methods:**

A retrospective review was performed on 47 patients that underwent percutaneous MR-guided focal cryoablation for biopsy-proven locally recurrent prostate cancer after primary radiotherapy. Preprocedural diagnostic and intraprocedural MR images were analysed to derive three-directional iceball margins. Local tumour progression after cryoablation was defined as evident tumour recurrence on follow-up MRI, positive MR-guided biopsy or biochemical failure without radiological evidence of metastatic disease.

**Results:**

Mean iceball margins were 8.9 mm (range −7.1 to 16.2), 10.1 mm (range 1.1–20.3) and 12.5 mm (range −1.5 to 22.2) in anteroposterior, left–right and craniocaudal direction respectively. Iceball margins were significantly smaller for tumours that were larger (*P* = .008) or located in the posterior gland (*P* = .047). Significantly improved local progression-free survival at 1 year post focal cryoablation was seen between patients with iceball margin >10 mm (100%), 5–10 mm (84%) and <5 mm (15%) (*P* < .001).

**Conclusions:**

Iceball margins appear to correlate with local outcome following MR-guided focal salvage prostate cryoablation. Our initial data suggest that freezing should be applied at minimum 5 mm beyond the border of an MR-visible recurrent prostate tumour for successful ablation, with a wider margin appearing desirable.

***Key points*:**

• *Shortest iceball margin most often occurred in anteroposterior direction*

• *Margins were smaller in tumours that were larger or posteriorly located*

• *Minimum iceball margin was a predictor of early local tumour progression*

• *A minimum 5-mm margin seems required for effective cryoablation of recurrent PCa*

## Introduction

Prostate cancer (PCa) recurrence following radiotherapy (RT) is not uncommon, with an estimated 10–60% of patients experiencing biochemical failure within 5–10 years after treatment [1, 2]. There has been no consensus on optimal management of this patient group with a majority of patients receiving androgen deprivation therapy (ADT) [3]. Treatment options with curative intent are limited and include salvage radical prostatectomy (RP), brachytherapy, high-intensity focused ultrasound and cryoablation [4].

Salvage RP is generally regarded as a challenging procedure to perform and has been associated with high complication risks [5]. Whole-gland salvage cryoablation has emerged as an accepted treatment alternative, with acceptable oncological outcomes and side effects [6, 7]. However, complications such as erectile dysfunction (72–100%), incontinence (2.6–73%) and rectal fistula (0–3.4%) may still exist [8, 9]. In an attempt to reduce treatment morbidity, several studies have been exploring the concept of partial cryoablation for biopsy-proven unilateral recurrences [9–11]. At the same time, advancements in prostate magnetic resonance (MR) imaging have enabled the accurate detection and localization of locally recurrent PCa after previous irradiation [12, 13]. Additionally, MRI can also be used to intraoperatively guide cryoablation procedures [14, 15], opening new possibilities for focal salvage approaches.

In a recent study the feasibility of MR-guided focal cryoablation was shown in ten patients with locally recurrent PCa after primary RT [16]. Although initial short-term results were promising, three patients developed local tumour recurrence at the site of ablation within 6–12 months after treatment and the authors concluded that these tumours were most probably undertreated. Findings from in vitro and in vivo animal studies on renal cryoablation have shown that lethal temperatures may be achieved at approximately 5–6 mm within the edge of the iceball [17, 18]. For prostate cryoablation, some authors have suggested freezing to be applied 2–3 mm beyond the tumour boundary to reach an end temperature of −20 °C, which has been associated with tissue necrosis [19]. However, limited clinical data is presently available verifying these recommendations with patient outcomes and a consensus on the margin needed for effective cryoablation of recurrent PCa has not been established. The purpose of the present study was to evaluate iceball margins after MR-guided focal salvage prostate cryoablation and determine the correlation with local outcome.

## Materials and methods

In July 2016, a total of 61 consecutive patients were identified that underwent MR-guided focal cryoablation for locally recurrent PCa at our institution between May 2011 and July 2015 and were at least 1 year after treatment. Ten patients were excluded because they had undergone previous salvage treatment. Additionally, three patients who underwent radical prostatectomy as primary treatment and one patient who was treated in two separate sessions were excluded. A total of 47 patients (median age 66 years, age range 52–79) underwent MR-guided focal cryoablation for local PCa recurrence after primary RT and were included in this retrospective study. Informed consent to use anonymized data for analysis was obtained in all patients.

Patients were eligible for focal cryoablation on the basis of biopsy-proven MRI-visible local PCa recurrence without radiologic evidence of metastatic disease. The final decision to perform cryoablation was made at the multidisciplinary board meeting. Focal cryoablation was performed at a median of 5 years (range 1–18) after primary RT. Prior to treatment, all patients underwent 3-T multi-parametric MR imaging (mpMRI) with a pelvic phased-array coil (*n* = 43) or endorectal coil (*n* = 4), consisting of T2-weighted (T2w), diffusion-weighted (DWI) and dynamic contrast-enhanced (DCE) imaging, to localize the recurrence and measure visible tumour volume as well as an abdominal and pelvic staging MRI to exclude nodal or bone marrow metastases. There were no restrictions related to pretreatment prostate specific antigen (PSA) level, Gleason score, prior history of ADT, tumour volume or local extent. The National Comprehensive Cancer Network (NCCN) guidelines were used to categorize patients into low-to-intermediate and high-risk groups [20]. High-risk status was assigned when any of the following criteria were met: cancer stage ≥ T3a, Gleason ≥8 or PSA ≥20 ng/ml. A summary of patient demographics is included in Table 1.

**Table 1:** Patient demographic data (*N* = 47)

	Median (range) or *n* (%)
Age (years)	66 (52–79)
PSA level (ng/ml)	4.9 (0.7–31.0)
Form of primary RT	
EBRT	29 (62)
Brachytherapy	17 (36)
EBRT + brachytherapy	1 (2)
Time to recurrence since primary RT (years)	5 (1–18)
Prior ADT use	
Yes	17 (36)
No	30 (64)
Gleason score of recurrence	
≤ 6	7 (15)
7	17 (36)
≥ 8	16 (34)
Unknown or undeterminable	7 (15)
Clinical stage	
T2a–c	35 (69)
T3b	12 (31)
NCCN risk category	
Low-to-intermediate	18 (38)
High	29 (62)
Prostate volume (ml)	23.5 (6.6–64.2)
Tumour localization	
Transition zone	11 (23)
Peripheral zone and/or seminal vesicles	36 (77)
Tumour volume (ml)	1.1 (0.2–11.8)

*EBRT* external beam radiotherapy, *ADT* androgen-deprivation therapy, *NCCN* National Comprehensive Cancer Network

### Cryoablation procedures

Patients were treated under general anaesthesia in a 1.5-T or 3-T MR system (Magnetom Avanto or Skyra, Siemens, Erlangen, Germany). All procedures were performed by one of two interventional radiologists (J.F. or S.J.) each with more than 6 years of experience in prostate interventions. A urethral warming catheter and rectal balloon were inserted to protect the urethra and rectal wall. A transperineal approach was used to place multiple MR-compatible cryoprobes (MRI IceSeed or IceRod, Galil Medical, Yokneam, Israel) centrally in the target region under real-time MR image guidance. Type, number and position of cryoprobes were chosen at the discretion of the performing physician. Cryoablation was performed using an MR-compatible cryoablation device (MRI-SeedNet; Galil Medical, Yokneam, Israel). Two 10-min freezing cycles separated by 2-min passive and 1-min active thaw were applied under continuous T1-weighted gradient echo MR monitoring [21, 22]. The intraprocedural images were used to modulate the size and shape of the iceball by regulating the gas flow to each individual cryoprobe so as to cover the entire tumour while avoiding contact between the iceball and rectal wall. Upon completion of the ablation, all cryoprobes were thawed and removed. All intraprocedural complications were documented. Postprocedural complication data, functional outcomes and quality-of-life metrics were not part of the present work and will be reported elsewhere.

### Image review and follow-up

All preprocedural and intraprocedural images were retrospectively reviewed by one prostate interventional radiologist (S.J.) using medical image visualization software (MeVisLab, Fraunhofer, Bremen, Germany). Treated tumours were annotated on the pretreatment diagnostic mpMRI using information from all available MR sequences to delineate the tumour boundary. Tumour locations were classified as anterior when more than 50% located in the ventral half of the prostate or posterior when located more than 50% in the dorsal half of the prostate.

**Fig. 1:**
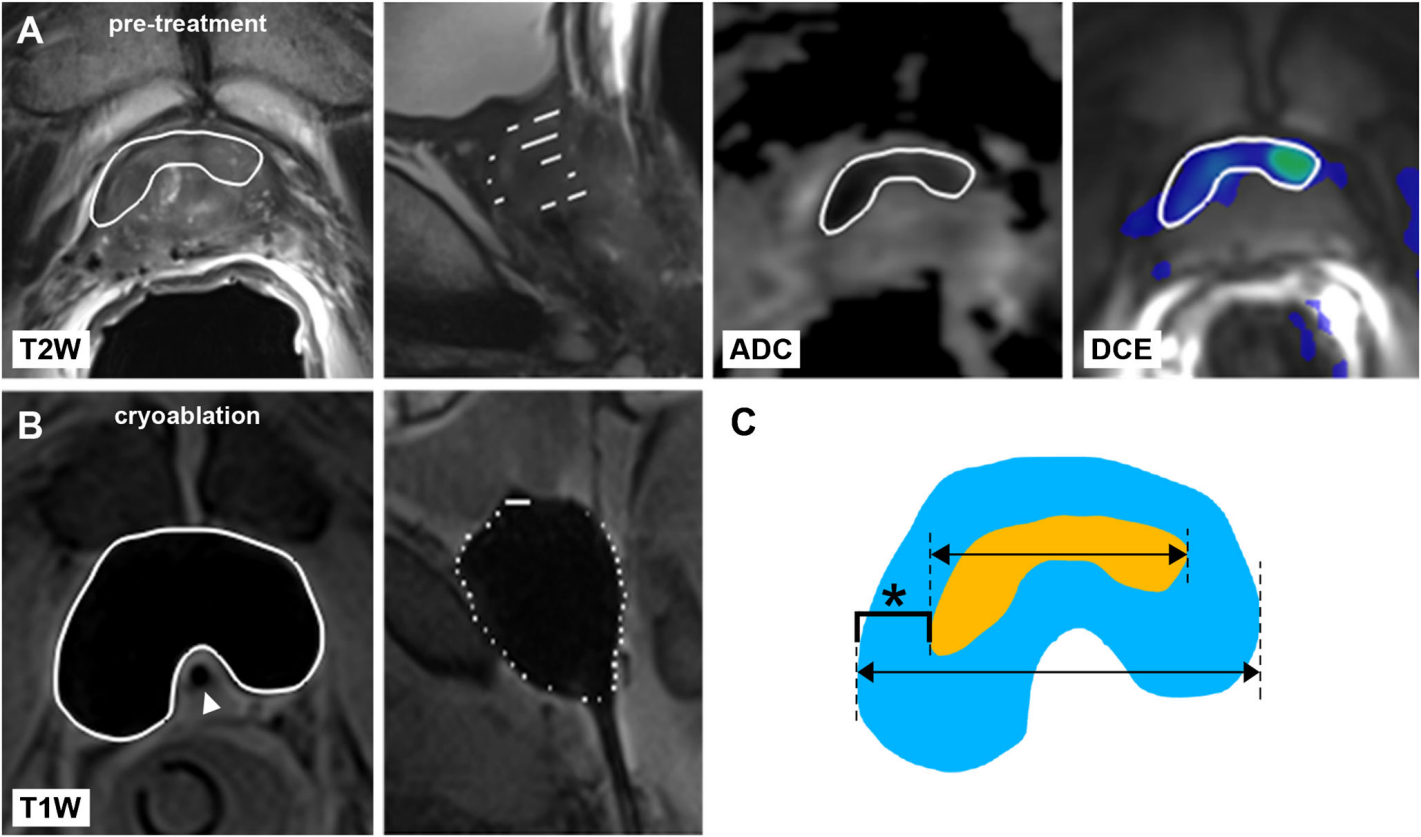
Annotation of **a** tumour (*outlined in white*) on pretreatment axial and sagittal T2-weighted, ADC and DCE prostate MR images and **b** corresponding iceball (*outlined in white*) at the end of the second freeze cycle on axial and sagittal intraprocedural T1-weighted images, with urethral warming catheter in situ (*arrowhead*). **c** The iceball margin (*asterisk*) was determined by subtracting the radii of the tumour (*yellow*) and iceball (*blue*) along each direction

Intraprocedural MR images obtained at the end of the second freeze cycle were used to annotate each corresponding iceball. MeVisLab software was used register each annotated iceball to the centre of the corresponding annotated tumour. Maximum tumour and corresponding iceball sizes were derived from the annotated regions in anteroposterior, left–right and craniocaudal direction as well as total tumour and iceball volumes. The iceball margin was then defined as the margin calculated by subtracting the iceball’s radius from the corresponding lesion radius in each direction (Fig. 1). The minimum iceball margin was defined as the shortest margin in each patient. Undertreated tumours were defined by a negative margin between the iceball and tumour. Finally, the position of the iceball relative to the tumour was recorded: a well-centred iceball covered the entire tumour in the axial plane at the centre of the lesion.

Postprocedural follow-up (median 24 months, range 3–42) consisted of regular urology visits with PSA monitoring at 1, 3, 6, 9 and 12 months after treatment, followed by 3- to 6-month intervals. In addition, all patients underwent follow-up prostate mpMRI at 3, 6 and 12 months after treatment. All follow-up images were reviewed by one of two prostate interventional radiologists (J.F. or S.J.). Any finding suspected of indicating residual or recurrent tumour was subsequently sampled by targeted in-bore MR-guided biopsy. Furthermore, all patients received targeted in-bore MR-guided biopsy directed at the ablation zone at 12 months post-treatment from October 2013 onwards. Local tumour progression after focal cryoablation was defined as evident tumour recurrence on follow-up MRI, positive MR-guided biopsy or biochemical failure according to Phoenix criteria of PSA nadir + 2 ng/ml [23] without radiological evidence of metastatic disease.

### Statistical analysis

Statistics were performed using SPSS (version 20.0). Data are reported as mean ± standard deviation unless indicated otherwise. Baseline parameters were compared between cases of local control and tumour progression using independent samples *t* test for normally distributed and Mann–Whitney *U* test for non-normally distributed data. Chi-square analysis was used for discrete data. Independent samples *t* test was used to compare iceball margins by tumour volume and location. Survival analysis was performed using the Kaplan–Meier method. Log-rank test was used to assess differences between survival curves. Cox proportional hazard univariable and multivariable models were used to assess pretreatment risk status, prior ADT use and tumour volume as potential confounders and determine whether iceball margins were independently associated with local progression. Potential prognostic factors with a *P* value less than 0.10 on univariable analysis were included in the multivariable model. Outcomes were reported as hazard ratios (HR) with 95% confidence intervals (CI) to demonstrate level of precision. *P* values less than 0.05 were considered statistically significant.

## Results

A total of 47 cryoablation procedures were successfully performed in 47 patients. A median of 3 cryoprobes (range 2–6) were used per patient. The median iceball volume was 27.7 ml (range 6.0–82.5). There were no intraprocedural complications. The median PSA nadir after focal prostate cryoablation was 0.6 ng/ml (range <0.02–40.0) and was reached at a median of 3 months (range 1–24) after treatment.

On follow-up, 24 of 47 patients (51%) were locally controlled. Local tumour progression occurred in 23 patients (49%), of whom 14 had a positive MR-guided prostate biopsy, seven experienced PSA failure without radiological evidence of distant disease and two patients had evident tumour recurrence on follow-up MRI but were spared targeted biopsy because of detection of nodal or bone metastases on the same scan. The median time between treatment and local tumour progression was 12 months (range 3–42). After multidisciplinary consensus seven patients with local tumour progression received repeat treatment with MR-guided cryoablation. Ten out of the 47 patients (21%) showed metastatic disease progression, of whom six were locally controlled and four patients also had evidence of local tumour progression.

The distribution of baseline parameters between cases of local control and tumour progression is shown in Table 2. A statistically significant difference was found in prior history of ADT administration between the stable and local progression group (5/24 vs. 11/23; *P* = .025). No difference was seen for pretreatment PSA level, Gleason score and clinical stage.

**Table 2:** Comparison of baseline parameters

	Local control (*N* = 24)	Local tumour progression (*N* = 23)	*P* value
PSA level	6.8 (0.7–31.0)	7.1 (1.1–21.0)	.566
Gleason score			.604
≤7	14 (58)	10 (43)	
>7	8 (33)	8 (35)	
Unknown	2 (8)	5 (22)	
Clinical stage			.154
T2a–c	20 (83)	15 (65)	
T3b	4 (17)	8 (35)	
Prior ADT use			.025
Yes	5 (21)	11 (48)	
No	19 (79)	12 (52)	

Values in parentheses are range or percentages

### Iceball margins

The mean iceball margins for focal cryoablation were 8.9 mm (range −7.1 to 16.2), 10.1 mm (range 1.1–20.3) and 12.5 mm (range −1.5 to 22.2) in anteroposterior, left–right and craniocaudal directions respectively. The mean minimum iceball margin per patient was 7.5 mm (range −7.1 to 14.2), with the shortest margin most often occurring in anteroposterior direction (49%). An iceball margin greater than 5 mm in each direction was achieved in 34/47 (72%) of patients. Negative margins occurred in three cases (6%). Iceballs were well centred in all but one patient. The one off-centre iceball occurred in a large tumour involving the peripheral zone and seminal vesicles. The iceball was found to be off-centre on retrospective review of the intraprocedural images because the insertion angle of the cryoprobes was incorrectly aligned with the tumour’s geometry, causing the iceball to be off-centre at the prostatic base and seminal vesicles.

**Fig. 2:**
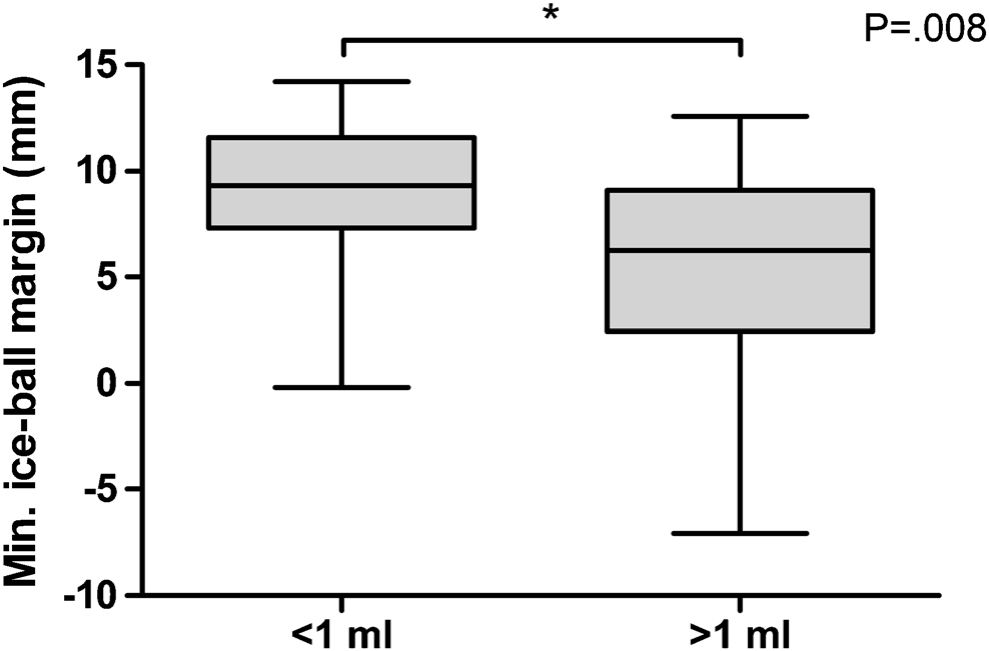
Minimum iceball margin stratified by tumour volume

**Fig. 3:**
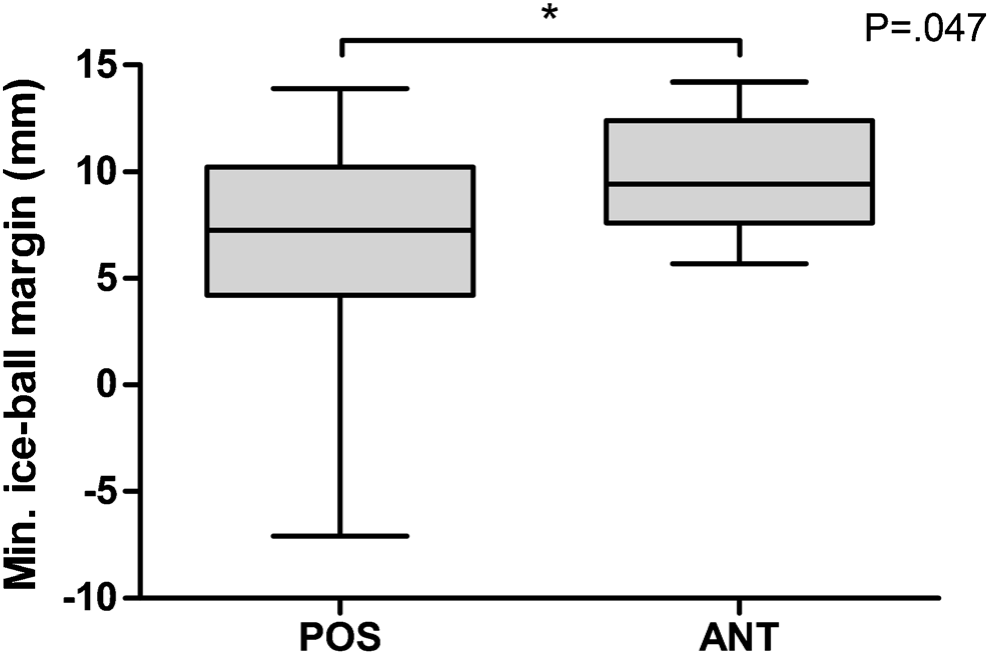
Minimum iceball margin by anterior or posterior tumour locations

When stratified by tumour volume and location, the average minimum iceball margin was significantly smaller for tumours larger than 1 ml compared to tumours 1 ml or smaller (5.8 ± 4.8 vs. 9.2 ± 3.4 mm; *P* = .008) (Fig. 2) and for posterior versus anterior tumours (6.8 ± 4.7 vs. 9.8 ± 2.7 mm; *P* = .047) (Fig. 3).

**Fig. 4:**
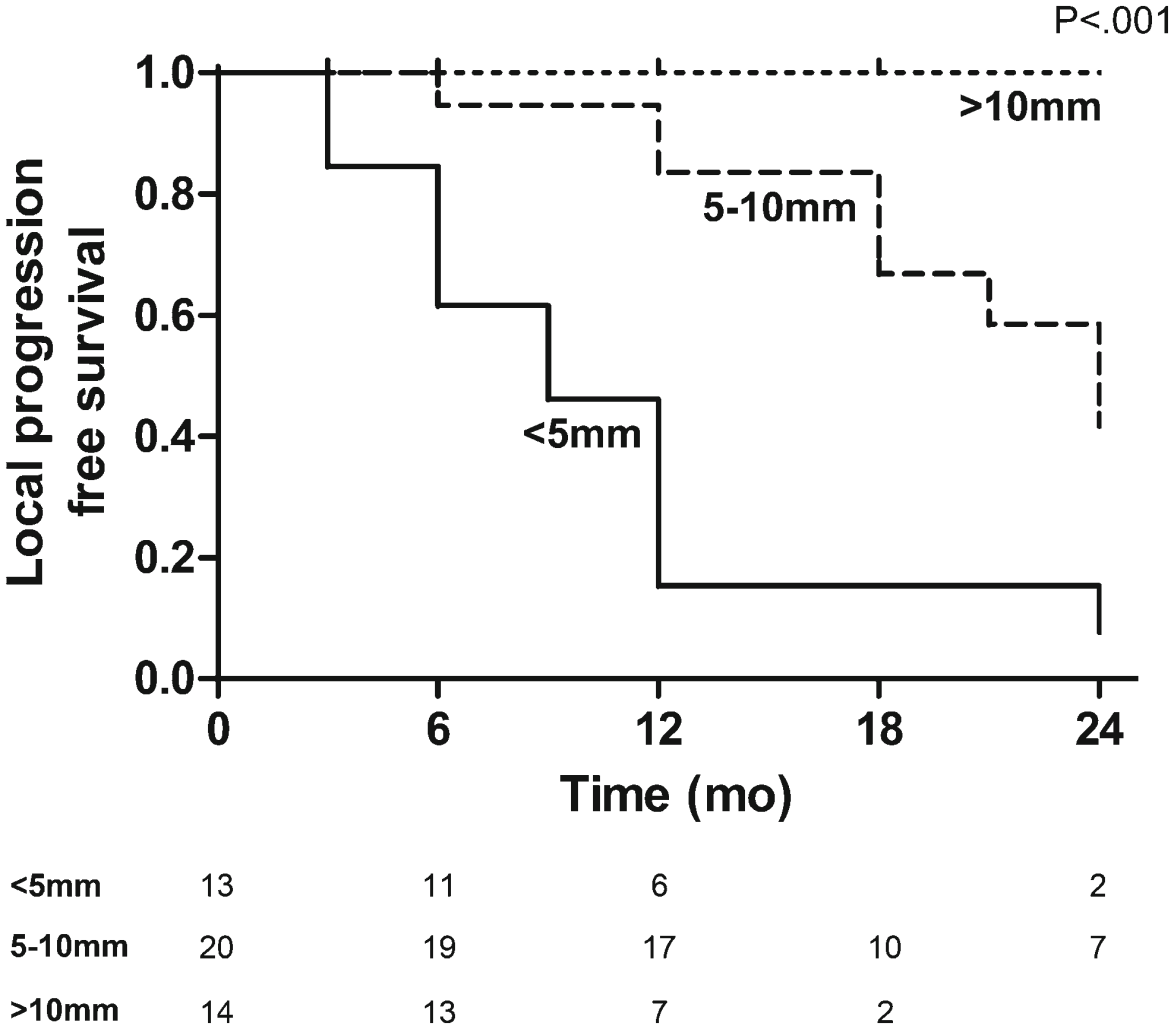
Local progression-free survival stratified by minimum iceball margin <5 mm, 5–10 mm and >10 mm, with corresponding numbers at risk

**Fig. 5:**
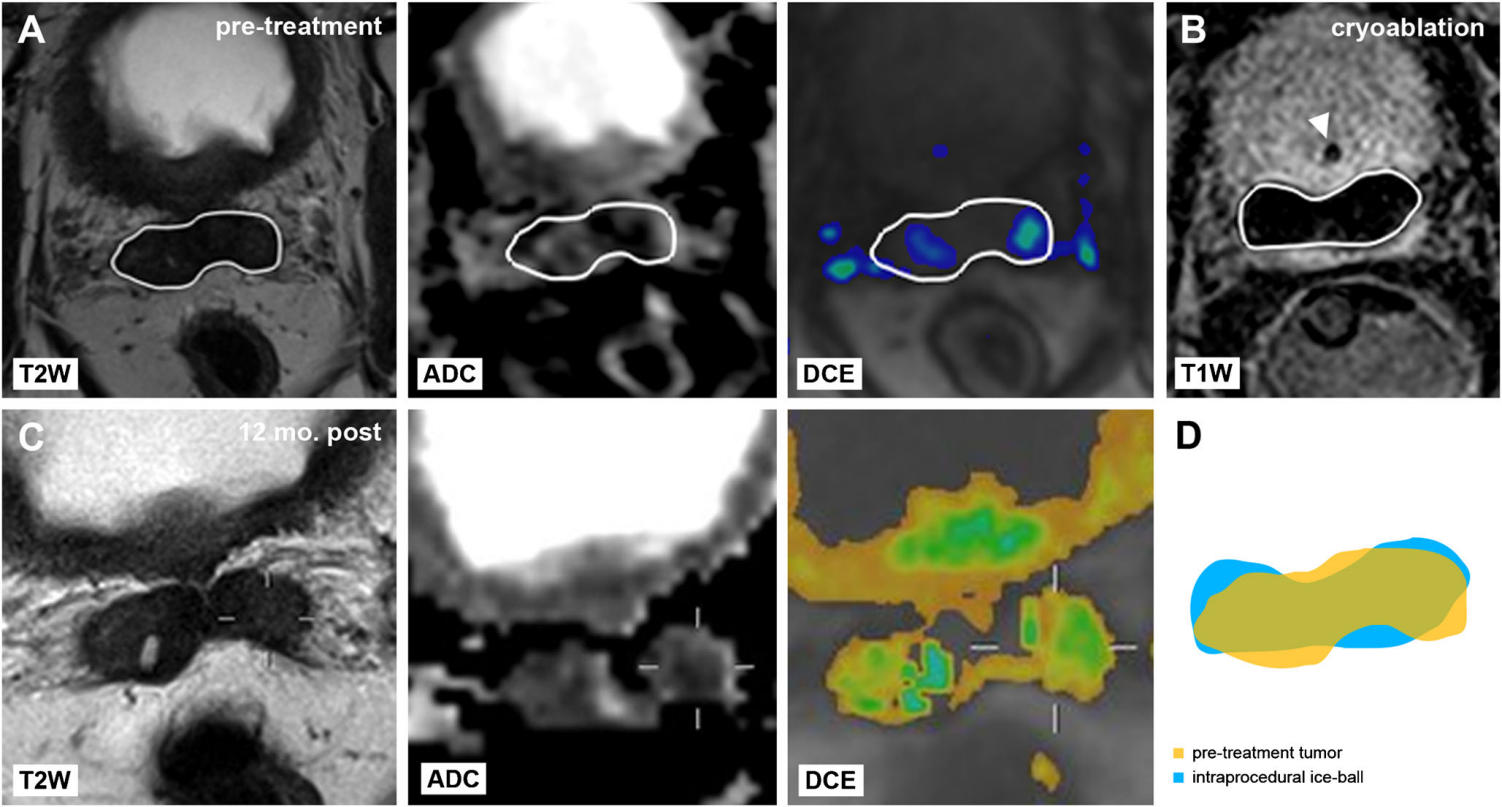
Representative case of local tumour progression after MR-guided focal salvage cryoablation. **a** Pretreatment axial T2-weighted, ADC and DCE images show a hypointense lesion with diffusion restriction and focal enhancement in the prostatic base extending into the seminal vesicles after primary radiotherapy. Local PCa recurrence was histologically confirmed with in-bore MR-guided biopsy. Annotated tumour boundary is overlaid (*white outline*). **b** Corresponding intraprocedural axial T1-weighted image during MR-guided cryoablation shows the final frozen zone at the end of the second freeze cycle, with annotated iceball boundary overlaid (*white outline*). Urethral warming catheter is indicated (*arrowhead*). **c** 12-month follow-up MRI shows hypointense signal on axial T2-weighted images with diffusion restriction and focal enhancement in the seminal vesicles. Local recurrence after cryoablation was confirmed on targeted in-bore MR-guided biopsy. **d** Retrospective review of this case showed a minimum iceball margin of −7.1 mm, with insufficient coverage in anteroposterior direction

**Fig. 6:**
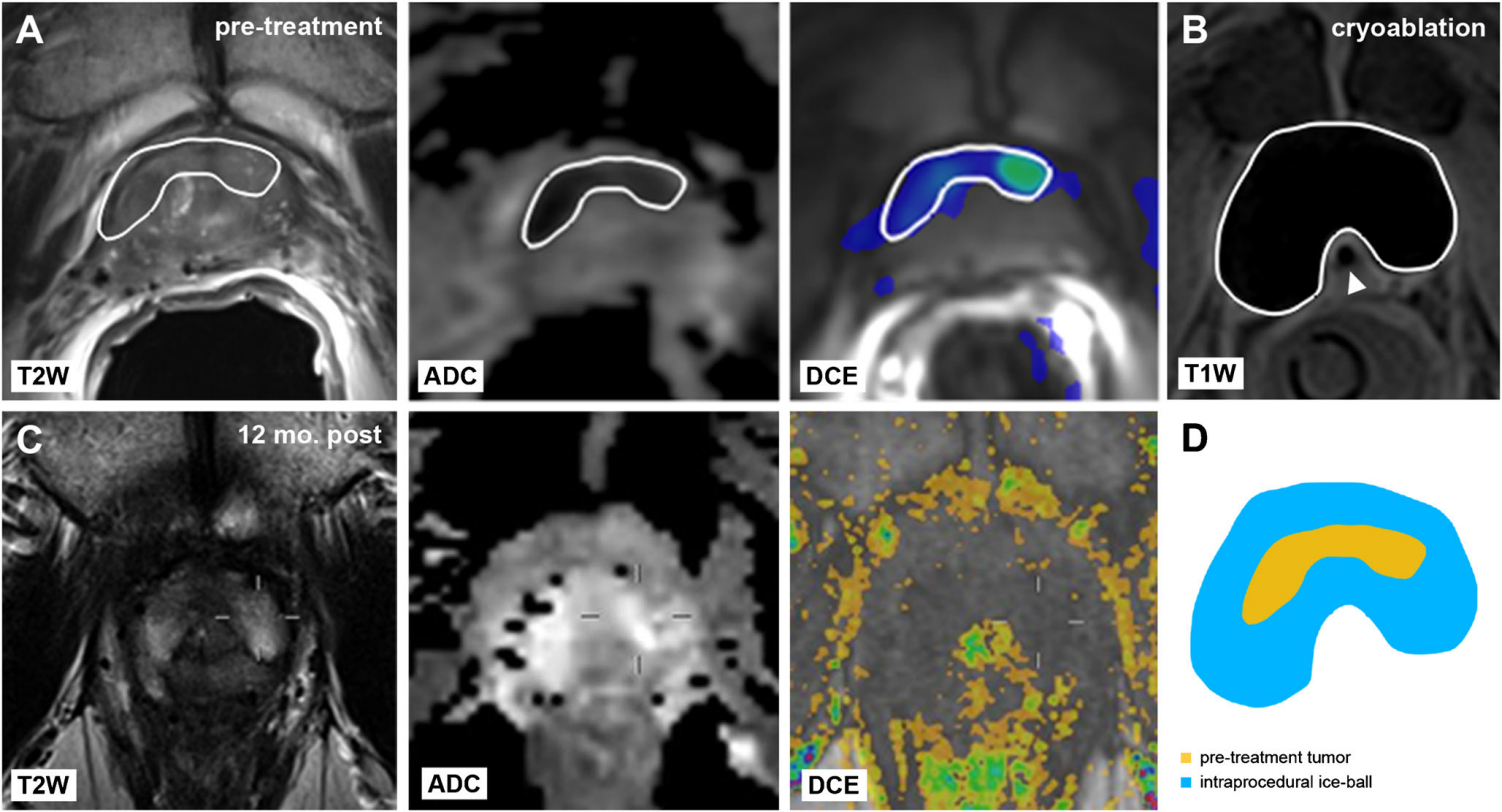
Representative case of local tumour control. **a** Pretreatment axial T2-weighted, ADC and DCE images demonstrate a histologically confirmed local PCa recurrence with diffusion restriction and focal enhancement in the ventral transition zone (*white outline*) after primary brachytherapy. **b** Intraprocedural axial T1-weighted image shows the final frozen zone (*white outline*) at the end of the second freeze cycle, with urethral warming catheter indicated (*arrowhead*). **c** 12-month follow-up MRI shows hyperintense signal on axial T2w images without diffusion restriction at the ventral transition zone. DCE images show a non-perfused area at the same location that coincides with the treated area. Targeted in-bore MR-guided biopsy directed at the ablation area showed no evidence of malignancy. **d** The retrospective review of this case showed adequate coverage in all directions, with the minimum iceball margin being 9.4 mm

On Kaplan–Meier analysis, significantly improved local tumour progression-free survival at 1 year after focal cryoablation was seen between patients with minimum iceball margin greater than 10 mm (100%), 5–10 mm (84%) and less than 5 mm (15%) (*P* < .001) (Fig. 4). Representative cases with and without local tumour progression after focal salvage cryoablation are shown in Figs. 5 and 6 respectively. Univariable and multivariable Cox regression analyses are shown in Table 3. Minimum iceball margin (*P* < .001), tumour volume (*P* = .005) and prior ADT use (*P* = .014) showed significant association with local outcome on univariable analysis. On multivariable analysis, minimum iceball margin (*P* = .004) and prior ADT use (*P* = .008) remained independent predictors of local tumour progression.

**Table 3:** Cox regression analysis of predictors of local progression

	Univariable	*P* value	Multivariable	*P* value
	HR (95% CI)		HR (95% CI)	
Min. iceball margin	0.85 (0.78–0.93)	<.001	0.85 (0.77–0.95)	.004
Tumour volume	1.25 (1.07–1.45)	.005	1.08 (0.91–1.28)	.40
Prior ADT use	0.35 (0.15–0.81)	.014	0.30 (0.12–0.73)	.008
NCCN risk category	1.67 (0.70–3.97)	.25	–	

## Discussion

The concept of focal therapy is to treat a tumour focus with an adequate margin while sparing as much surrounding healthy tissue as possible [24]. In cryoablation, the lethal temperature threshold is generally regarded to lie between −20 °C to −40 °C, depending on freezing rate, freeze duration and the repetition of freeze-thaw cycles [25]. With lethal temperatures residing several millimetres behind the leading edge of the freezing process [26], it is imperative to achieve a definitive margin with sufficient ice extending beyond the tumour border to ensure effective tissue necrosis throughout the entire target region. For renal cryoablation, studies have recommended freezing to be applied for approximately 5–6 mm beyond the tumour border to achieve suitably cold end temperatures [18, 27, 28]. In contrast, another study suggested that for liver cryoablation with a single cryoprobe a margin up to 1 cm might not be sufficient [29], but the synergistic effect of multiple cryoprobes was not studied [26, 30]. Furthermore, it is unclear how findings from other areas of application, i.e. renal or liver cryoablation, apply to the prostate salvage setting. Adding to this, uncertainty exists in predicting tumour borders from MRI with respect to the true histological tumour boundary. For primary PCa, one study has recently shown that imaging-derived tumour boundaries from prostate mpMRI tend to underestimate the histologically determined tumour volume at prostatectomy [31]. The authors concluded that a treatment margin extending 9 mm around an MRI visible tumour would be required to ensure treatment of the complete tumour volume during focal ablative therapy [32]. A similar study investigating the accuracy of MR-based prostate tumour delineations for radiotherapy planning recommended a margin of 5 mm to achieve adequate coverage [33]. In the present work, MR imaging-derived iceball margins were correlated with local outcome after focal salvage prostate cryoablation. A high rate of early local tumour progression (85% at 1 year) was noted when the iceball margin around an MRI-visible recurrent prostate tumour were less than 5 mm. These initial clinical data suggest that a minimum 5-mm margin would be required for effective focal salvage prostate cryoablation, with a wider margin appearing desirable.

A notable finding was that the iceball margins varied significantly with tumour location. In the posterior area of the prostate it can be particularly challenging to achieve sufficient ice coverage extending beyond the tumour boundary because of its close proximity to the rectum. During ablation, adjacency of the iceball to the rectal wall was actively monitored in the intraoperative MR images and freeze intensity was modulated accordingly to avoid unwanted damage to the rectal wall. Also, the rectum was actively warmed with warm saline using a rectal balloon, which may explain the lower iceball margins achieved in posterior tumours. For these tumour localizations, critical preoperative assessment of the ability to achieve adequate ablation margins should be performed in each individual case. Additionally, displacement of the rectum using hydrodissection may be considered to obtain increased spacing between the prostate and rectal wall and allow for a wider ablation margin.

There also was a significant difference in the average iceball margin depending on tumour volume. However, substantial overlap can be seen between the margins achieved in the larger (>1 ml) and smaller (<1 ml) lesions and successful ablations were achieved in tumours up to 3.3 ml in volume when a 5-mm margin was achieved. Likewise, local tumour progression occurred after ablation of lesions as small as 0.3 ml when the margin was insufficient in one direction. Together, these findings suggest that even though it is more difficult to achieve wide ablation margins in larger tumours, successful ablation can be achieved. In addition, in small tumours, the need for an adequate iceball margin remains important.

Of baseline parameters, prior history of ADT use was significantly different between cases of local control and tumour progression and demonstrated significant association with local tumour progression on multivariate analysis as well. Although large randomized studies have shown ADT to improve cancer-specific and overall survival when given neoadjuvantly to primary radiation [34, 35], the exact role of ADT before salvage treatment has not been well defined. An early study has investigated the effect of the combination of androgen deprivation with salvage surgery in patients with radiorecurrent prostate cancer and suggested that patients in whom an initial trial of ADT failed were poor candidates for salvage prostatectomy [36]. Similarly, for whole-gland salvage prostate cryoablation one study has recently shown that patients receiving ADT before salvage cryoablation had worse 5-year biochemical progression-free survival [37]. One explanation is that the initiation of ADT before salvage cryoablation may have concealed rising PSA levels, masking micrometastatic disease progression. Consequently, at the time of salvage cryoablation patients with systemic disease may be unintentionally subjected to local salvage treatment.

Possibly, the latter is corroborated by the substantial proportion of patients diagnosed with metastatic disease progression during follow-up of the present study (21%). All but two of these instances occurred within the first 12 months after focal salvage treatment and metastatic work-up may have been false negative in these patients, with current imaging techniques having limited sensitivity [38]. Improved metastatic screening using new contrast agents such as iron nanoparticles-enhanced MR lymphography [38, 39] or gallium-68-PSMA PET-CT [40] to specifically evaluate lymph node status may be imperative to better select suitable candidates for local salvage treatment.

The most important limitation of our study was that iceball margins were only calculated in three directions and assumed the iceball to be well centred with respect to the tumour to register the annotated regions. In some patients, margins may not necessarily have been symmetrical and there could be a sufficient margin on one side but an insufficient margin on the other side when a tumour was not centrally located within the ablation zone. Ideally, all pretreatment images would be exactly matched to the intraprocedural data to provide an improved assessment of the 3D ablation margin. In our experience, however, direct registration of the image sets will not necessarily be accurate because of prostate deformation from cryoprobe insertion or movement of the entire gland due to the rectal balloon, and adequate methods to address these errors are needed to provide a more detailed characterization of the true ablation margin in future studies.

Other limitations to this work include its retrospective nature and short- to intermediate-term follow-up. Also, there is currently no universally accepted definition of tumour recurrence following local prostate salvage treatments. The Phoenix definition is the most commonly reported criterion in this setting and in one study was found to more accurately predict local cancer recurrence following prostate cryoablation than American Society for Therapeutic Radiotherapy and Oncology (ASTRO) criteria [41]. Imaging follow-up of the prostate after focal therapy using mpMRI has also been increasingly applied, with functional techniques showing promise to detect local PCa recurrence after therapy [42]. In this study, both biochemical as well as mpMRI and biopsy findings were integrated into a combined definition of local tumour progression. Ultimately, a standardized definition would need to be identified to facilitate direct comparison of studies evaluating prostate focal ablation techniques. Finally, heterogeneity can be seen in our patient cohort with respect to presalvage PSA levels, Gleason scores and clinical stage. Our findings require validation on multivariable analysis in more patients and with longer follow-up to come to definitive conclusions.

In conclusion, iceball margins appear correlated with local outcome following MR-guided focal salvage prostate cryoablation. Our initial data suggest that a minimum margin of 5 mm should be achieved around an MR-visible recurrent tumour for successful ablation, with a wider margin being desirable. Adequate margins may be more difficult to achieve in tumours that are larger and located posteriorly, which may be an important factor to consider when selecting candidates for focal salvage treatment after previous RT.
